# The role of the systemic inflammatory response index in predicting postoperative atrial fibrillation

**DOI:** 10.1590/1806-9282.20240783

**Published:** 2025-03-31

**Authors:** Muhammet Hüseyin Erkan, Ömer Faruk Rahman, Firat Durna

**Affiliations:** 1 Nevşehir State Hospital, Department of Cardiovascular Surgery – Nevşehir, Turkey.; 2 İzmir Bakırçay University, Department of Cardiovascular Surgery – İzmir, Turkey.

**Keywords:** Coronary artery bypass, Biomarkers, Atrial fibrillation

## Abstract

**OBJECTIVE:**

The aim of the present study was to determine the effect of systemic inflammatory response index in predicting the development of postoperative atrial fibrillation after isolated coronary artery bypass grafting (CABG).

**METHODS:**

The study was conducted at Nevşehir State Hospital, a secondary healthcare center. Patients who underwent elective isolated CABG between October 2018, when the first open-heart surgery was performed at our center, and December 2022 were included. The patients’ data were retrospectively reviewed and recorded.

**RESULTS:**

A total of 66 patients were included in the study (mean age: 64.14±8.59 years). Of these patients, 44 (66.7%) were male and 22 (33.3%) were female. Postoperative atrial fibrillation was present in 22 (33.3%) patients. Although the systemic inflammatory response index and systemic immune-inflammatory index values were higher in the patient group that developed postoperative atrial fibrillation, no significant difference was noted (p>0.050). Additionally, age and a family history of coronary artery disease were identified as important factors that significantly affected the development of postoperative atrial fibrillation (p=0.048 and p=0.01, respectively).

**CONCLUSION:**

To the best of our knowledge, no study has investigated the relationship between postoperative atrial fibrillation and the systemic inflammatory response index. Although the present study did not find a statistically significant difference, our findings support the role of inflammation in predicting postoperative atrial fibrillation.

## INTRODUCTION

artery bypass grafting (CABG) is a widely practiced procedure for coronary artery disease (CAD) treatment. However, postoperative atrial fibrillation (PoAF) after this surgical intervention is a serious complication occurring in 20–40% of patients^1^. PoAF significantly increases the risk of stroke, morbidity, and mortality, leading to longer hospital stays and higher healthcare costs^2^. Predicting and preventing this complication is crucial for improving postoperative care.

Inflammation is known to play a significant role in the pathophysiology of PoAF that develops after CABG. The inflammatory response is a systemic process triggered by surgical trauma and cardiopulmonary bypass (CPB). It has been reported that levels of inflammatory markers such as interleukin-6 (IL-6) and C-reactive protein increase in the postoperative period, and this increase is associated with the development of atrial fibrillation (AF)^3^. This inflammatory response increases the risk of AF by exacerbating myocardial damage and electrical imbalances.

Assessing the systemic immune response is considered a crucial step in predicting and managing postoperative complications following CABG. Changes in the functions of immune cells, such as dendritic cells and monocytes, have been associated with adverse outcomes, including prolonged intensive care unit stays and PoAF^4^. The responses of these immune cells after surgery correlate with the increase in inflammatory markers. The systemic inflammatory response index (SIRI) is a biomarker calculated using the counts of neutrophils, monocytes, and lymphocytes^5^. Moreover, studies have found that SIRI is associated with cardiovascular diseases and an increased risk of supraventricular tachycardia^6^.

The systemic immune-inflammatory index (SII) is another biomarker reflecting the degree of inflammation and immune response. The SII uses three blood cell subtypes (neutrophils, lymphocytes, and platelets) to indicate the balance between inflammation and immune response^7^. Studies have shown that high SII levels can predict various cardiovascular events^8^.

This study aimed to investigate the relationship between the degree of inflammation, as measured by the new biomarkers SIRI and SII, and PoAF in patients undergoing CABG.

## METHODS

### Patients

The study commenced after receiving approval from the Ethics Committee of Nevşehir Hacı Bektaş Veli University (approval no. 2022.07.73). This study was designed to include patients who underwent elective isolated CABG at Nevşehir State Hospital between October 2018, when the hospital performed its first open-heart surgery, and December 2022. Exclusion criteria were as follows: patients with the presence of preoperative atrial fibrillation or other arrhythmias, decompensated heart failure, and comorbid hormonal disorders that could trigger arrhythmias and those who underwent emergency CABG and scheduled additional cardiac surgical procedures. Nine patients did not meet the inclusion criteria, and therefore were excluded from the study. Ultimately, a total of 66 patients were included in the study.

PoAF was defined as atrial fibrillation lasting longer than 5 min and confirmed by a 12-lead electrocardiogram at any point until discharge following surgical treatment. The demographic data, clinical characteristics, echocardiographic and angiographic findings, operational variables, hemogram, and biochemistry parameters of the included patients were retrieved from their medical records. The SIRI and the SII were calculated using these parameters.

### Statistical analysis

The data were analyzed using the IBM SPSS version 22.0 program. Normally distributed quantitative data were examined using independent two-sample t-tests for binary groups, and non-normally distributed quantitative data were analyzed using the Mann-Whitney U test. For the analysis of categorical data in binary groups, Yates's correction, Pearson's chi-square test, and Fisher's exact test were employed. Factors influencing the development of PoAF in patients were examined using a logistic regression analysis. The analysis results were presented as mean±standard deviation, median (minimum–maximum), and frequency (percentage). A p<0.05 was considered statistically significant.

## RESULTS

The mean age of the 66 patients who met the inclusion criteria for the study was 64.14±8.59 years. Of these patients, 44 (66.7%) were male and 22 (33.3%) were female. PoAF was present in 22 (33.3%) patients. The distribution of the demographic characteristics of the patients according to the PoAF status is presented in Table 1. Significant differences were observed between the groups in terms of age and a family history of CAD, p=0.048 and p=0.01, respectively.

**Table 1:** Distribution of the demographic characteristics of patients according to groups.

Demographic characteristics		PoAF		Test ist.	p
		None (n=44)	Present (n=22)		
Age		62.89±9.61	66.64±5.47	-2.018	0.048^t^
BMI		28.34±3.48	29.33±3.15	-1.121	0.266^t^
Gender					
	Female	12 (27.3)	10 (45.5)	1.44	0.230^y^
	Male	32 (72.7)	12 (54.5)		
Chronic disease*					
	Hypertension	33 (76.7)	16 (80)	5.965	0.310^pk^
	Hyperlipidemia	35 (81.4)	13 (65)		
	Diabetes mellitus	29 (67.4)	15 (75)		
	PAH	3 (7)	0 (0)		
	COPD	11 (25.6)	2 (10)		
Family history of CAD					
	Absent	0 (0)	4 (18.2)	–	0.010^f^
	Present	44 (100)	18 (81.8)		
Smoking					
	Absent	13 (29.5)	10 (45.5)	1.009	0.315^y^
	Present	31 (70.5)	12 (54.5)		
Alcohol use					
	Absent	34 (77.3)	18 (81.8)	–	0.759^f^
	Present	10 (22.7)	4 (18.2)		
Preoperative medication use*					
	Beta blockers	35 (79.5)	19 (86.4)	4.492	0.722^pk^
	ACE inhibitors	11 (25)	8 (36.4)		
	ARBs	3 (6.8)	2 (9.1)		
	Calcium channel blockers	5 (11.4)	4 (18.2)		
	Clopidogrel	5 (11.4)	2 (9.1)		
	Statin	15 (34.1)	4 (18.2)		
	Acetylsalicylic acid	42 (95.5)	20 (90.9)		
LVEF %		60 (35±65)	59 (45±70)	473.500	0.881^m^

* Multiple responses,

t independent two-sample t-test,

y Yates's correction,

pk Pearson's chi-square test,

f Fisher's exact test,

m Mann-Whitney U test. PoAF: postoperative atrial fibrillation; BMI: body mass index; PAH: pulmonary arterial hypertension; COPD: chronic obstructive pulmonary disease; CAD: coronary artery disease; ACE: angiotensin-converting enzyme; ARBs: angiotensin receptor blockers; LVEF: left ventricular ejection fraction.

While examining descriptive statistics, it was observed that in patients who developed PoAF, the levels of postoperative urea, sodium, hemoglobin, hematocrit (HT), red cell distribution width (RDW), white blood cell (WBC) count, neutrophil count, monocyte count, mean platelet volume (MPV), and platelet distribution width (PDW) were higher, whereas the levels of creatinine, potassium, calcium, aspartate aminotransferase (AST), alanine aminotransferase (ALT), lymphocyte count, and platelet count were lower (Table 2). However, the difference in postoperative laboratory parameters was not statistically significant (p>0.05). Similarly, the SII and SIRI values were higher in the group with PoAF, but no statistically significant difference was observed (p>0.05).

**Table 2:** Distribution of operative variables and postoperative laboratory parameters according to groups.

Postoperative laboratory parameters and operative variables		PoAF		Test ist.	p
		None (n=44)	Present (n=22)		
Urea		35 (20±63)	38 (22±67)	442.500	0.572^m^
Creatinine		0.81 (0.06±1.48)	0.68 (0.46±1.48)	349.500	0.067^m^
Sodium		139.5 (134±150)	140 (134±153)	411.500	0.320^m^
Potassium		4.41±0.5	4.2±0.52	1.588	0.117^t^
Calcium		8.1 (7.1±11.4)	7.95 (6.8±10.8)	382.500	0.166^m^
AST		35 (22±214)	34.5 (19±260)	443.000	0.577^m^
ALT		26 (9±65)	16.5 (8±68)	353.500	0.076^m^
Hemoglobin		9.3 (6.4±12.9)	9.47 (7.56±105)	444.000	0.586^m^
HT		28.59±3.35	29.28±3.93	-0.750	0.456^t^
RDW		14.1 (11.9±167)	14.55 (12.4±141)	475.000	0.903^m^
WBC		10.4 (4.9±30.4)	12.2 (2.9±24.8)	418.500	0.373^m^
Neutrophil		9.2 (3.77±25.2)	10.5 (2.5±21.1)	432.000	0.479^m^
Lymphocyte		0.82 (0.29±3.81)	0.74 (0.34±2.79)	467.000	0.817^m^
Monocyte		0.32 (0.01±2.06)	0.5 (0.02±1.44)	367.000	0.111^m^
Platelet		173.5±54.33	162±46.14	0.850	0.398^t^
MPV		9.13±0.8	9.38±1	-1.102	0.275^t^
PDW		16.2 (11.8±20.8)	16.75 (13.8±26.5)	387.000	0.187^m^
SII		1,646.33 (154.08±6,828.8)	1,802.71 (490.65±7,041.15)	452.000	0.663^m^
SIRI		61.47 (4.84±374.98)	71.92 (5.16±318.24)	445.000	0.596^m^
RCA bypass					
	No	18 (40.9)	9 (40.9)	–	1.000^f^
	Yes	26 (59.1)	13 (59.1)		
Number of anastomoses					
	1	5 (11.4)	1 (4.5)	1.656	0.799^pk^
	2	8 (18.2)	4 (18.2)		
	3	21 (47.7)	13 (59.1)		
	4	9 (20.5)	4 (18.2)		
	5	1 (2.3)	0 (0)		
Duration of CPB (min)		111 (27±182)	115 (0±155)	481.000	0.967^m^
Cross-clamp time (min)		55.7±21.5	57.23±21.06	-0.273	0.786^t^

m Mann-Whitney U test,

t independent two-sample t-test,

pk Pearson's chi-square test,

f Fisher's exact test. PoAF: postoperative atrial fibrillation; ALT: alanine aminotransferase; AST: aspartate aminotransferase; HT: hematocrit; RDW: red cell distribution width; WBC: white blood cell; MPV: mean platelet volume; PDW: platelet distribution width; SII: systemic immune-inflammatory index; SIRI: systemic inflammatory response index; RCA: right coronary artery; CPB: cardiopulmonary bypass.

When evaluated based on the number of anastomoses, it was observed that triple bypass was performed in 34 patients (51.5%). The number of patients who underwent right coronary artery bypass was 39 (59.1%). In this study, the median CPB time was 113.5 min, and the median cross-clamp time was 58.5 min. No statistically significant difference was noted between the groups in terms of operative variables (p>0.05). The distribution of the operative variables for each group is presented in Table 2.

The role of various variables in predicting PoAF was evaluated using univariate and multivariate logistic regression models (Table 3). According to the results obtained, parameters such as age, hypertension, preoperative beta-blocker drug use, CPB time, cross-clamp time, and SII and SIRI values did not have a statistically significant effect on the development of PoAF (p>0.050).

**Table 3:** Investigation of factors affecting postoperative atrial fibrillation status.

All factors affecting	Univariate		Multivariate	
	OR (95%CI)	p	OR (95%CI)	p
Age	1.058 (0.989±1.132)	0.100	1.065 (0.991±1.145)	0.085
HT (reference: none)	0.889 (0.279±2.836)	0.842	0.699 (0.189±2.586)	0.591
Beta-blocker drug use (reference: none)	1.629 (0.393±6.744)	0.501	1.866 (0.395±8.824)	0.431
Duration of CPB (min)	1.000 (0.985±1.015)	0.976	0.989 (0.953±1.025)	0.542
Cross-clamp time (min)	1.003 (0.979±1.028)	0.782	1.019 (0.959±1.082)	0.543
SII	1.000 (0.999±1.000)	0.794	1.000 (0.999±1.000)	0.438
SIRI	1.001 (0.994±1.007)	0.803	1.001 (0.993±1.009)	0.840

OR: odds ratio; CI: confidence interval; SII: systemic immune-inflammatory index; SIRI: systemic inflammatory response index; CPB: cardiopulmonary bypass; HT: hematocrit.

## DISCUSSION

The present study examined the relationship between PoAF that develops after CABG and the SIRI. To the best of our knowledge, ours is the first study conducted in Turkey in this regard. Our findings suggest that PoAF is a common complication following CABG and can be associated with specific risk factors.

**Figure 1:**
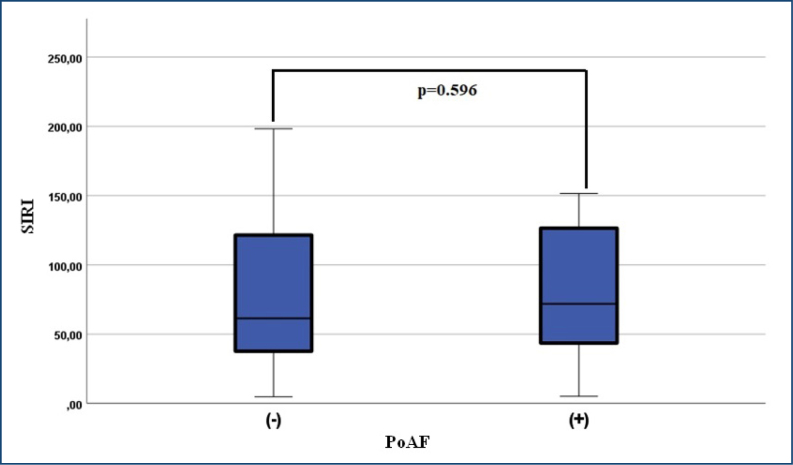
Comparison of changes in systemic inflammatory response index scores between postoperative atrial fibrillation-positive and postoperative atrial fibrillation-negative patients. PoAF: postoperative atrial fibrillation; SIRI: systemic inflammatory response index.

The SIRI is a composite index derived from the absolute counts of three distinct inflammatory cells: neutrophils, monocytes, and lymphocytes^9,10^. Elevated SIRI values have been linked to an increased risk of myocardial infarction and higher overall mortality rates^11^. Moreover, the SIRI has been identified as an independent predictor of functional outcomes following intracerebral hemorrhage and acute ischemic stroke^12,13^. Although not statistically significant in our study, we observed higher SIRI values in patients who developed PoAF, suggesting an increased level of inflammation (Figure 1).

In our study, we also evaluated the SII, calculated based on peripheral neutrophil, platelet, and lymphocyte counts, which provides an integrated measurement of inflammatory and prothrombotic responses^14,15^. Higher SII levels indicate increased systemic inflammation and immune activation. Moreover, elevated SII levels, along with increased atrial inflammatory infiltration and fibrosis, represent prothrombotic conditions that accelerate microthrombosis and atrial ischemia, thereby contributing to the formation of PoAF^16,17^. Although we observed higher SII values in patients who developed PoAF in our study, statistical significance was not reached. This could be attributed to the limited sample size in our study.

Advanced age is the main risk factor for PoAF, and the risk of PoAF approximately doubles for every 10-year increase in age^18^. In our study, age showed a significant relationship with the development of PoAF. This may be attributed to changes in leukocyte telomere length associated with age and alterations in the inflammatory response due to mitochondrial dysfunction^19^. Additionally, while the present study showed a significant relationship between a family history of CAD and the development of PoAF, no such relationship has been observed in previous studies^20^.

The present study discovered that the levels of urea, sodium, hemoglobin, HT, RDW, WBC, neutrophils, monocytes, MPV, and PDW during the postoperative period were higher in patients who developed AF; however, the differences were not statistically significant. Elevated monocyte levels support inflammation, and previous studies have found higher levels of monocyte chemoattractant protein-1 in the epicardial adipose tissue of patients who developed PoAF^21^. Furthermore, similar to our study, in some studies, patients who developed PoAF were found to have higher levels of monocytes despite statistically insignificant elevated levels of WBC count, RDW, and MPV^22,23^.

Electrolyte imbalances, particularly low serum potassium and calcium levels, are known to have an impact on the development of PoAF^24,25^. Similar to the literature, serum potassium and calcium levels were found to be lower in the present study. However, the differences between these parameters were not statistically significant.

### Strengths and limitations

This study has some strengths and limitations. First, our sample size is limited, and the study was designed retrospectively. Second, inflammatory markers were evaluated only once, and changes during the process were not monitored. However, the design of this study was good, and this is the first study in the literature to investigate the relationship between PoAF that develops after CABG and the SIRI.

## CONCLUSION

The results of this study indicate that age and a family history of CAD play a significant role in the development of PoAF, but other laboratory and inflammatory parameters do not have a significant impact. Although our findings support the potential role of inflammation in predicting PoAF, studies with larger samples are needed in this field. A more comprehensive examination of inflammatory markers and systemic immune response will allow the prevention and better management of PoAF.
